# Biangular-Combined Vegetation Indices to Improve the Estimation of Canopy Chlorophyll Content in Wheat Using Multi-Angle Experimental and Simulated Spectral Data

**DOI:** 10.3389/fpls.2022.866301

**Published:** 2022-04-15

**Authors:** Weiping Kong, Wenjiang Huang, Lingling Ma, Chuanrong Li, Lingli Tang, Jiawei Guo, Xianfeng Zhou, Raffaele Casa

**Affiliations:** ^1^Key Laboratory of Quantitative Remote Sensing Information Technology, Aerospace Information Research Institute, Chinese Academy of Sciences, Beijing, China; ^2^State Key Laboratory of Remote Sensing Science, Aerospace Information Research Institute, Chinese Academy of Sciences, Beijing, China; ^3^School of Marine Technology and Geomatics, Jiangsu Ocean University, Lianyungang, China; ^4^College of Life Information Science and Instrument Engineering, Hangzhou Dianzi University, Hangzhou, China; ^5^Department of Agricultural and Forestry Sciences (DAFNE), Università degli Studi della Tuscia, Viterbo, Italy

**Keywords:** winter wheat, multi-angle hyperspectral remote sensing, canopy chlorophyll content, biangular combination, crop phenotype

## Abstract

Canopy chlorophyll content (CCC) indicates the photosynthetic functioning of a crop, which is essential for the growth and development and yield increasing. Accurate estimation of CCC from remote-sensing data benefits from including information on leaf chlorophyll and canopy structures. However, conventional nadir reflectance is usually subject to the lack of an adequate expression on the geometric structures and shaded parts of vegetation canopy, and the derived vegetation indices (VIs) are prone to be saturated at high CCC level. Using 3-year field experiments with different wheat cultivars, leaf colors, structural types, and growth stages, and integrated with PROSPECT+SAILh model simulation, we studied the potential of multi-angle reflectance data for the improved estimation of CCC. The characteristics of angular anisotropy in spectral reflectance were investigated. Analyses based on both simulated and experimental multi-angle hyperspectral data were carried out to compare performances of 20 existing VIs at different viewing angles, and to propose an algorithm to develop novel biangular-combined vegetation indices (BCVIs) for tracking CCC dynamics in wheat. The results indicated that spectral reflectance values, as well as the coefficient of determination (*R*^2^) between mono-angular VIs and CCC, at back-scattering directions, were mostly higher than those at forward-scattering directions. Mono-angular VIs at +30° angle, were closest to the hot-spot position in our case, achieved the highest *R*^2^ among 13 viewing angles including the nadir observation. The general formulation for the newly developed BCVIs was BCVI_VI_ = *f* × VI_(θ1)_ − (1 − *f*) × VI_(θ2)_, in which the VI was used to characterize chlorophyll status, while the subtraction of VI at θ1 and θ2 viewing angles in a proportion was used to highlight the canopy structural information. From our result, the values of the θ1 and θ2 around hot-spot and dark-spot positions, and the *f* of 0.6 or 0.7 were found as the optimized values. Through comparisons revealed that large improvements on CCC modeling could be obtained by the BCVIs, especially for the experimental data, indicated by the increase in *R*^2^ by 25.1–51.4%, as compared to the corresponding mono-angular VIs at +30° angle. The BCVI_MCARI[705,750]_ was proved to greatly undermine the saturation effect of mono-angular MCARI[705,750], expressing the best linearity and the most sensitive to CCC, with *R*^2^ of 0.98 and 0.72 for simulated and experimental data, respectively. Our study will eventually have extensive prospects in monitoring crop phenotype dynamics in for example large breeding trials.

## Introduction

Canopy chlorophyll content (CCC) is defined as the total amount of chlorophyll present in the canopy per unit ground area. The CCC, as the product of leaf chlorophyll content (LCC) and leaf area index (LAI), is capable of indicating the overall characteristics of plant assemblages, to avoid the deficiency of LCC, which mainly involves the information of individual plant conditions. It is an important phenotypic trait for crop breeding since it can represent the plant’s capacity to intercept and use sunlight through photosynthesis. Also, it is a key factor influencing crop biological function with consequences on many aspects, including crop phenotypes and plant stress, as well as crop quality and yield (Merzlyak et al., 1999; Huang et al., 2011; Gutierrez et al., 2015). In addition, CCC is proven to be very sensitive to N availability in the soil (Hinzman et al., 1986), thus precise monitoring of CCC plays an important role in optimizing N fertilizer strategy, and consequently, obtaining a higher yield, and at the same time, avoiding the waste of resources and the pollution of farmland ecosystem in the context of precision agriculture. In the past decades, the significance of CCC for crop growth and development status and agricultural management has motivated the interest and substantial efforts of researchers on high-throughput determination of crop CCC using remote-sensing data (Broge and Leblanc, 2001; Broge and Mortensen, 2002; Li et al., 2016), and has provided the rationale for improving our capability to remotely measure it at the field or larger scales.

Conventionally, CCC estimation was mostly based on spectral reflectance acquired from a near nadir direction. Several optical indices have arisen in the literatures and have been proven to be well-correlated with vegetation chlorophyll content (Daughtry et al., 2000; Sims and Gamon, 2002; Gitelson et al., 2006; Wu et al., 2008). As the significant relationship between leaf nitrogen and chlorophyll (Li et al., 2013), a series of nitrogen indices [e.g., Nitrogen Reflectance Index (NRI), Normalized Difference of the Double-peak Areas (NDDA), Ratio Vegetation Index (RVI), Normalized Difference Vegetation Index green-blue (NDVI_g–b_)] were proposed for tracking nitrogen changes according to spectral features of chlorophyll (Bausch and Duke, 1996; Hansen and Schjoerring, 2003; Xue et al., 2004; Feng et al., 2014), and in turn, they have also been investigated to assess crop chlorophyll status (Li et al., 2016). However, most of these vegetation indices (VIs) were prone to suffering from saturation (Sims and Gamon, 2002; Haboudane et al., 2004), thus, reducing their sensitivity to high chlorophyll content. Researchers have been hard at work finding ways to cope with this issue, although quite difficult, if not impossible, to achieve. One of the approaches is to use the red-edge bands to take the place of red bands partly due to the unique characteristics and potential of the red edge region for chlorophyll estimation (Blackburn, 1998). For example, Gitelson and Merzlyak (1994) focused on improving the commonly and widely used Normalized Difference Vegetation Index (NDVI) and Simple Ratio (SR) and proposed the NDVI[705,750] and SR[705,750]. Wu et al. (2008) developed the Modified Chlorophyll Absorption Ratio Index (MCARI[705,750]) and MCARI/Optimized Soil-adjusted Vegetation Index (MCARI/OSAVI[705,750]) based on previously published MCARI and MCARI/OSAVI (Daughtry et al., 2000), by taking into account the effect of quick saturation at the red band. Ground truth validation showed an appropriate result for high chlorophyll content estimation in winter wheat and maize. However, these VIs were calculated from the nadir spectral reflectance, which is mainly contributed by the upper leaves of the canopy (Li et al., 2013), making it very difficult to depict the chlorophyll information over the whole canopy, especially for the complicated canopies that vary in vegetation types, canopy structures, background contributions, etc. (Leblanc et al., 1997). Moreover, the lack of expression of information on the geometric structures and the shaded parts of vegetation canopy would limit the use of nadir-based VIs for accurate determination of chlorophyll status when upscaling to canopy level, and then, hardly an adequate description of characteristics of plant communities.

A possible alternative and complementary method to minimize these limitations presented above is the exploitation of multi-angle remote-sensing technology. Multi-angle observations contain much more information than the simple nadir observation since they capture the information of an area of interest from several different angles. It is demonstrated that multi-angle canopy reflectance has the ability of assessing three-dimensional canopy structure that is poorly detected by the nadir alone (Chen et al., 2003; Brown de Colstoun and Walthall, 2006), so they are expected to provide the possibility to evaluate CCC more accurately for crops. There have been studies showing that off-nadir spectral sensing generated more effective VIs for monitoring leaf biological parameters when compared to the nadir direction (Stagakis et al., 2010; Zhang et al., 2021). Several recent studies that used multi-angle spectral data also focused on developing new multi-angular VIs aiming to obtain truly better vegetation variables inversions (e.g., LAI, leaf nitrogen content, and water use efficiency) than the conventional VIs (Hasegawa et al., 2010; Wu et al., 2010; He et al., 2016; Zhang et al., 2021). Indeed, these VIs have enriched the methodology for vegetation parameters estimation with remote-sensing technology. Nevertheless, few researchers have reported the construction of multi-angular VIs for crop CCC retrieval.

In recent years, some multi-angle observing data are already available from sensors mounted on different remote sensing platforms. Compared to airborne and spaceborne platforms, such as Multi-angle Imaging Spectroradiometer (MISR), the Compact High-Resolution Imaging Spectrometer (CHRIS), the ground-based goniometers, are used more extensively, since they can measure vegetation canopy at higher spatial resolution, as well as extremely various directions, by adjusting angular sampling and viewing height in a very flexible way. In addition, the PROSPECT+SAILh (PROSAIL) model describes how light propagation within vegetation canopy and has been successfully used before to develop and test various VIs for estimating leaf parameters for multiple types of vegetation including wheat (Haboudane et al., 2004; Wu et al., 2008; Zhou et al., 2019). It allows for the simulation of reflectance at arbitrary viewing and illumination geometries and a set of leaf and canopy parameters, providing another convenient avenue to create multi-angle spectral data and characterize different traits for a wheat phenotype.

The main purpose of this study is to propose the BCVI that includes abundant chlorophyll and structural information of plant communities, yet, resistant to saturation limits, using multi-angle spectral data, then, benefits the high-throughput and nondestructive determination of crop CCC compared to the conventional mono-angular VIs. The analyses are based on a simulated canopy multi-angle hyperspectral reflectance dataset produced by PROSAIL model in combination with real ground measured data collected from 3-year field campaigns. The study is composed of three phases: (1) to analyze the characteristics of angular anisotropy in spectral reflectance; (2) to examine the performances of previously published mono-angular VIs in CCC estimation and identify the VIs that is sensitive to CCC of winter wheat; (3) to develop the new BCVIs by coupling spectral and angular information and compare their performances with the corresponding mono-angular VIs, to evaluate the improvement of CCC estimation when the multi-angle observation was used.

## Materials and Methods

### Experimental Design

**FIGURE 1:**
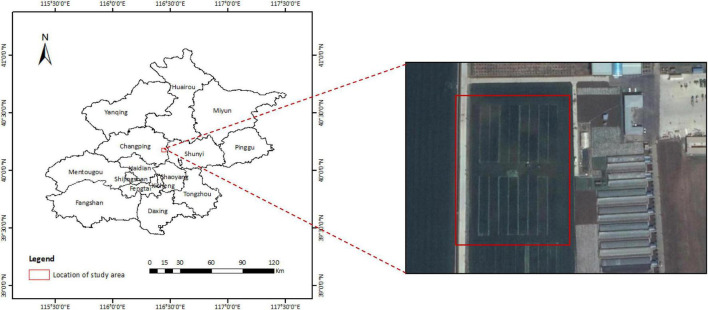
The overview map of study area (cited from Kong et al., 2021).

The experiments were conducted over 3 years (2004, 2005, and 2007) at Xiaotangshan National Precision Agriculture Experimental Site (116°120′E, 40°13.20′N), in Changping district, Beijing, China (Figure 1). This experimental site has been operational since 2001 and used for precision agriculture research. The crop selected in this study was winter wheat, which was cultivated in silty clay soil with sufficient water supply and uniform nutrient management. The nutrients of soil in the topsoil layer (0–.20 m depth) were as follows: 1.42–2.2% of organic matter, 117.6–129.1 mg/kg of available potassium, and 20.1–55.4 mg/kg of available phosphorus. Information on different measurement times, wheat cultivars, leaf colors, leaf structural types, and sampling dates were summarized in Table 1. All cultivars were sown with a row space of 25 cm, each cultivar was planted in a plot and repeated three times. A total of 60 datasets, including canopy multi-angle spectral reflectance and corresponding CCC, were collected during the 3 years.

**TABLE 1:** Different measurement times, wheat cultivars, leaf colors, leaf structural types, and sampling dates for the experiments.

Year	Wheat cultivar	Leaf color	Leaf structural type	Sampling date
2004	Laizhou 3279	Dark green	Erective	Stem elongation (Z34), booting (Z47), heading (Z59)
	Linkang 2	Dark green	Loose	
	Jing 411	Light green	Erective	
	9507	Light green	Loose	
2005	Nongda 3291	Dark green	Erective	Stem elongation (Z34), booting (Z47), heading (Z59)
	Jingdong 8	Dark green	Middle	
	Linkang 2	Dark green	Loose	
	Lumai 21	Light green	Erective	
	Jingwang 10	Light green	Middle	
	9507	Light green	Loose	
2007	Laizhou 3279	Dark green	Erective	Stem elongation (Z31), stem elongation (Z34), booting (Z47), heading (Z59), milk-filling (Z73)
	I-93	Dark green	Erective	
	Linkang 2	Dark green	Loose	
	Jing 411	Light green	Erective	
	Jing 9428	Light green	Loose	
	9507	Light green	Loose	

### *In situ* Measurements

#### Measurement of Canopy Multi-Angle Spectral Reflectance

Canopy multi-angle spectral reflectance was measured in each plot using an ASD FieldSpec 3 spectrometer (Analytical Spectral Devices, Boulder, CO, United States), with a 25° field-of-view fiber optics, under clear sky conditions between 11:00 and 13:00 (Beijing local time) when minimum variations in solar view angle occur. The instrument records spectral radiance with a sampling interval of 1.4 nm and a resolution of 3 nm between 350 and 1,050 nm, and a sampling interval of 2.0 nm and a resolution of 10 nm between 1,000 and 2,500 nm. It was held on a rotating bracket to enable spectral measurements of the same target from different angles in a short time. Canopy multi-angle spectral measurements were conducted in the solar principal plane (constructed by the direction of incident direct sunlight, and the direction of the normal to surface target) at different viewing zenith angles (θ). A total of 13 viewing angles varied from −60° to +60° with 10° incremental step (i.e., θ = 0°, ±10°, ±20°, ±30°, ±40°, ±50°, and ±60°), where a positive angle refers to the back-scattering direction (the side facing away from the sun), a negative angle refers to the forward-scattering direction (the side facing into the sun). The nadir (i.e., θ = 0°) spectral measurements were made at a height of approximately 1.3 m above the canopy top. A white Spectralon (Labsphere, Inc., NH, United States) reference panel was used under the same illumination conditions to convert the spectral radiance to reflectance before and after canopy spectral measurements. Twenty scans were performed and averaged to obtain canopy spectral reflectance per viewing angle. More detailed information about the multi-angle spectral measurements can be found in previous studies (Wu et al., 2010; Huang et al., 2011).

#### Determination of Canopy Chlorophyll Content

Four 1-m consecutive rows of wheat in the plot, within the footprint of canopy multi-angle reflectance acquisitions, were harvested by cutting off the aboveground portions, then, put in cooled black plastic bags and transported to the laboratory to measure the biological parameters. Leaves that fully expanded and showed homogenous color, as well as no visible sign of damage, were sampled from top to bottom of the canopy. Two leaf disks (about 0.25 cm^2^) were cut-off from each leaf sample. One part of the disks were used for the chlorophyll extraction, which was carried out by immersing and grinding the disk in 10 mL aqueous acetone/distilled water buffer solution (80:20, volume proportion). After storing the solution in darkness for more than 24 h, the absorbance was measured with a UV-VIS spectrophotometer (Perkin-Elmer, Lambda 5, Waltham, MA, United States) at 645 and 663 nm wavelengths. Leaf chlorophyll *a* and chlorophyll *b* content (mg/L) were determined using Equations 1, 2 (Lichtenthaler, 1987). Another part of leaf disks was weighted after drying in an oven at 80°C for 48 h to determine leaf dry weight (DW, g), and then, used to compute leaf mass per area (LMA, g/cm^2^), defined as the ratio between leaf dry weight and leaf area (Poorter et al., 2009). The LAI measurement was conducted by the laboratory analysis, 10% of all the sampled leaves were taken as a subsample for leaf area measurement using a Li-Cor 3100 area meter (Lincoln, NB, United States), and the weight of leaves was recorded to scale up to the LAI of the 1 m^2^ area. The CCC (μg/cm^2^) was calculated as the product of LCC (μg/cm^2^) and LAI, as shown in Equation 6.


(1) $$\text{LCCa}\left(\text{mg}/\text{L}\right)=12.25A_{663}-2.79A_{645}$$

(2) $$\text{LCCb}\left(\text{mg}/\text{L}\right)=21.50A_{645}-5.10A_{663}$$

(3) $$\begin{array}{c} \text{LCCa}\left(\text{mg}/\text{g}\right)=\left[\text{LCCa}\left(\text{mg}/\text{L}\right)\times {\text{V}}_{\text{T}}\left(\text{ml}\right)\right]/\\ \;\left[\text{DW}\left(\text{g}\right)\times 1,000\right] \end{array}$$

(4) $$\begin{array}{c} \text{LCCb}\left(\text{mg}/\text{g}\right)=\left[\text{LCCb}\left(\text{mg}/\text{L}\right)\times {\text{V}}_{\text{T}}\left(\text{ml}\right)\right]/\\ \;\left[\text{DW}\left(\text{g}\right)\times 1,000\right] \end{array}$$

(5) $$\begin{array}{c} \text{LCC}\left(\mu g/{\text{cm}}^{2}\right)=\left[\text{LCCa}\left(\text{mg}/\text{g}\right)+\text{LCCb}\left(\text{mg}/\text{g}\right)\right]\\ \;\;\times \text{LMA}\left(\text{g}/{\text{cm}}^{2}\right)\times 1,000 \end{array}$$

(6) $$\text{CCC}\left(\mu \text{g}/{\text{cm}}^{2}\right)=\text{LCC}\left(\mu \text{g}/{\text{cm}}^{2}\right)\times \text{LAI}$$

where A_645_ and A_663_ are the absorbances of extract solution at wavelength 645 and 663 nm, respectively; LCCa is leaf chlorophyll *a* content, LCCb is leaf chlorophyll *b* content, V_T_(ml) is the volume of extract solution.

### PROSPECT+SAILh Model Simulation

In order to evaluate whether multi-angle observations can lead to the improved estimation of CCC, PROSAIL radiative transfer model, a coupled model of the leaf optical model PROSPECT and the canopy bidirectional reflectance model SAILh, was used for simulation of canopy multi-angle reflectance and sensitivity analysis of VIs. At the leaf level, PROSPECT model simulates leaf reflectance and transmittance between 400 and 2,500 nm at 1 nm increment, as a function of a series of biochemical parameters, including leaf mesophyll structure parameter (N), LCC, leaf carotenoid content (Car), leaf brown pigment content (C_brown_), leaf equivalent water thickness (C_w_), and leaf dry matter content (C_m_). The SAILh model has the capacity to simulate canopy multi-angle spectral reflectance, which is described as a function of LAI, average leaf angle (ALA), hot-spot parameter (hspot), soil moisture parameter (psoil), a fraction of diffuse incident radiation (skyl), and the parameters controlled the view-sensor-illumination geometry, i.e., solar zenith angle, view zenith angle, and the relative azimuth angle between the sun and sensor. The combined PROSPECT+SAILh model has been extensively used in a large number of studies and applications (Jacquemoud et al., 2009).

To perform the PROSAIL simulation, we set the LCC ranged from 25 μg/cm^2^ to 100 μg/cm^2^ in steps of 5 μg/cm^2^, whilst the LAI ranged from 1 to 8 in steps of 0.5, based on the field measured data regarding the wheat investigated in this study. The CCC was the product of the model input parameters LCC and LAI. The solar zenith angle was set to 30°, the values of view zenith angles were varied from 0° to 60° by changing the observation angle in 10° increments, as well as the relative azimuth angles between the sun and the sensor was set to 0° (corresponding to the back-scattering directions) and 180° (corresponding to the forward-scattering directions), which were all consistent with the field measurements. Input parameter C_brown_ was assigned a value of 0 since there were no brown leaves observed in the wheat canopy after visual inspection. Other input variables were either determined at the averaged values in accordance with the experimental plots or taken from the published literatures (Haboudane et al., 2004; Yu et al., 2014). A dataset of 3,120 canopy multi-angle reflectance simulations was generated by running PROSAIL model using a random combination of the input parameters (Table 2).

**TABLE 2:** Input parameters of PROSAIL model.

Parameters	Units	Values	Steps
*PROSPECT model*			
Leaf mesophyll structure parameter (N)	–	1.55	–
Leaf chlorophyll content (LCC)	μg/cm^2^	25–100	5
Leaf carotenoid content (Car)	μg/cm^2^	10	–
Leaf brown pigment content (C_brown_)	μg/cm^2^	0	–
Leaf equivalent water thickness (C_w_)	cm	0.013	–
Leaf dry matter content (C_m_)	g/cm^2^	0.0045	–
*SAILh model*			
Leaf area index (LAI)	m^2^/m^2^	1–8	0.5
Average leaf angle (ALA)	Degree	Spherical	–
Hot-spot parameter (hspot)	–	0.15	–
Soil moisture parameter (psoil)	–	1	–
Fraction of diffuse incident radiation (skyl)	–	0.23	–
Solar zenith angle	Degree	30	–
View zenith angle	Degree	0–60	10
Relative azimuth angle between the sun and sensor	Degree	0–180	180

### Mono-Angular and Biangular-Combined Vegetation Indices

Canopy spectral reflectance measured from different viewing angles was processed and analyzed as an individual dataset in this study. A total of 20 published VIs that were previously proposed for leaf chlorophyll and nitrogen estimates were selected. They were grouped into chlorophyll indices and nitrogen indices (Table 3). On one hand, these VIs were calculated from spectral reflectance obtained at a given viewing angle among 13 viewing angles, referred to as mono-angular VIs, then were tested for the potential of CCC estimation. On the other hand, we established a series of BCVIs based on the VIs shown in Table 3. The formula is given in Equation 7. The values of each VI at all the possible two-angle observations, selected from 13 viewing zenith angles between −60° and +60°, were combined in form of subtraction, with a parameter “*f*” changing from 0 to 1 at a step of 0.1 was used as an adjusting factor, resulting in 858 combinations of viewing angles and adjusting factor values. The BCVI built by a given VI was referred to as BCVI_VI_. In the selection of the VI used in the BCVI, the VI should be highly sensitive to the dynamics of chlorophyll. So, the VIs that achieved better results in quantifying chlorophyll content at mono-angular observations (shown in Figure 4 below) were chosen. Additionally, the value of *f* represented the proportion of VI at one angle (referred to as VI_(θ1)_), and the value of (1-*f*) represented the proportion of VI at the second angle (referred to as VI_(θ2)_). The difference of VI between the two angles was used to strengthen the canopy structural trait of the crop. As a consequence, this construction algorithm is expected to allow the CCC estimation with high accuracy when using the developed BCVI. All the calculations were implemented using MATLAB 8.3 (The MathWorks, Inc., Natick, MA, United States).


(7) $${\text{BCVI}}_{\text{VI}}=f\times {\text{VI}}_{\left(\theta 1\right)}-\left(1-f\right)\times {\text{VI}}_{\left(\theta 2\right)},f=0.1,0.2,\cdots ,1$$

**TABLE 3:** Published vegetation indices used in the analyses.

Vegetation index	Formula	References
*Chlorophyll indices*		
PSNDa (pigment specific simple ratio for chlorophyll *a*)	$\frac{R_{800}-R_{680}}{R_{800}+R_{680}}$	Blackburn, 1998
PSNDb (pigment specific simple ratio for chlorophyll *b*)	$\frac{R_{800}-R_{635}}{R_{800}+R_{635}}$	Blackburn, 1998
NDVI[705,750] (normalized difference vegetation index using 705 and 750 nm bands)	$\frac{R_{750}-R_{705}}{R_{750}+R_{705}}$	Gitelson and Merzlyak, 1994
SR[705,750] (simple ratio using 705 and 750 nm bands)	$\frac{R_{750}}{R_{705}}$	Gitelson and Merzlyak, 1994
CI_*green*_ (chlorophyll index using green band)	$\frac{R_{790}}{R_{550}}-1$	Gitelson et al., 2006; Clevers and Kooistra, 2012
CI_*rededge*_ (chlorophyll index using red edge band)	$\frac{R_{790}}{R_{710}}-1$	Gitelson et al., 2006; Clevers and Kooistra, 2012
MCARI (modified chlorophyll absorption ratio index)	$\left[\left(R_{700}-R_{670}\right)-0.2\left(R_{700}-R_{550}\right)\right]\left(\frac{R_{700}}{R_{670}}\right)$	Daughtry et al., 2000
MCARI[705,750] (modified chlorophyll absorption ratio index using 705 and 750 nm bands)	$\left[\left(R_{750}-R_{705}\right)-0.2\left(R_{750}-R_{550}\right)\right]\left(\frac{R_{750}}{R_{705}}\right)$	Wu et al., 2008
MCARI/OSAVI (MCARI/optimized soil-adjusted vegetation index)	$\frac{\left[\left(R_{700}-R_{670}\right)-0.2\left(R_{700}-R_{550}\right)\right]\left(\frac{R_{700}}{R_{670}}\right)}{\left(1+0.16\right)\left(R_{800}-R_{670}\right)/\left(R_{800}+R_{670}+0.16\right)}$	Rondeaux et al., 1996; Daughtry et al., 2000
MCARI/OSAVI[705,750] (MCARI/OSAVI using 705 and 750 nm bands)	$\frac{\left[\left(R_{750}-R_{705}\right)-0.2\left(R_{750}-R_{550}\right)\right]\left(\frac{R_{750}}{R_{705}}\right)}{\left(1+0.16\right)\left(R_{750}-R_{705}\right)/\left(R_{750}+R_{705}+0.16\right)}$	Wu et al., 2008
TCARI (transformed chlorophyll absorption ratio index)	$3\left[\left(R_{700}-R_{670}\right)-0.2\left(R_{700}-R_{550}\right)\left(\frac{R_{700}}{R_{670}}\right)\right]$	Haboudane et al., 2002
TCARI/OSAVI (TCARI/optimized soil-adjusted vegetation index)	$\frac{3\left[\left(R_{700}-R_{670}\right)-0.2\left(R_{700}-R_{550}\right)\left(\frac{R_{700}}{R_{670}}\right)\right]}{\left(1+0.16\right)\left(R_{800}-R_{670}\right)/\left(R_{800}+R_{670}+0.16\right)}$	Haboudane et al., 2002
TCARI/OSAVI[705,750] (TCARI/OSAVI using 705 and 750 nm bands)	$\frac{3\left[\left(R_{750}-R_{705}\right)-0.2\left(R_{750}-R_{550}\right)\left(\frac{R_{750}}{R_{705}}\right)\right]}{\left(1+0.16\right)\left(R_{750}-R_{705}\right)/\left(R_{750}+R_{705}+0.16\right)}$	Wu et al., 2008
TVI (triangular vegetation index)	0.5[120(*R*_750_−*R*_550_)−200(*R*_670_−*R*_550_)]	Broge and Leblanc, 2001
MTVI1 (modified TVI)	1.2[1.2(*R*_800_−*R*_550_)−2.5(*R*_670_−*R*_550_)]	Haboudane et al., 2004
REP (red edge position)	$700+40\times \frac{\left(R_{670}+R_{780}\right)/2-R_{700}}{R_{740}-R_{700}}$	Clevers and Kooistra, 2012
*Nitrogen indices*		
NDVI_*g–b*_ (normalized difference vegetation index using green and blue bands)	$\frac{R_{573}-R_{440}}{R_{573}+R_{440}}$	Hansen and Schjoerring, 2003
NRI (nitrogen reflectance index)	$\frac{R_{570}-R_{670}}{R_{570}+R_{670}}$	Bausch and Duke, 1996
NDDA (normalized difference of the double-peak areas)	$\frac{R_{755}+R_{680}-2\times R_{705}}{R_{755}-R_{680}}$	Feng et al., 2014
RVI (ratio vegetation index for nitrogen)	$\frac{R_{810}}{R_{560}}$	Xue et al., 2004

### Data Analysis

To investigate the angular anisotropy in spectral reflectance, the green, red, red edge, and near-infrared (NIR) bands (represent by 550, 680, 705, and 750 nm, respectively) were chosen because of their widespread use in deriving chlorophyll-related indices (Daughtry et al., 2000; Wu et al., 2008). We computed the normalized reflectance at all viewing zenith angles by normalizing the nadir reflectance as a reference for the above representative bands. Its formula was as follows:


(8) $$\text{Normalized}\mathrm{reflectance}={\text{R}}_{\left(\theta \right)}/{\text{R}}_{\left(\mathrm{Nadir}\right)}$$

where R_(θ)_ and R_(Nadir)_ indicate the spectral reflectance obtained from a given viewing zenith angle among the 13 viewing angles between −60° and +60° and the nadir observation, respectively.

**FIGURE 2:**
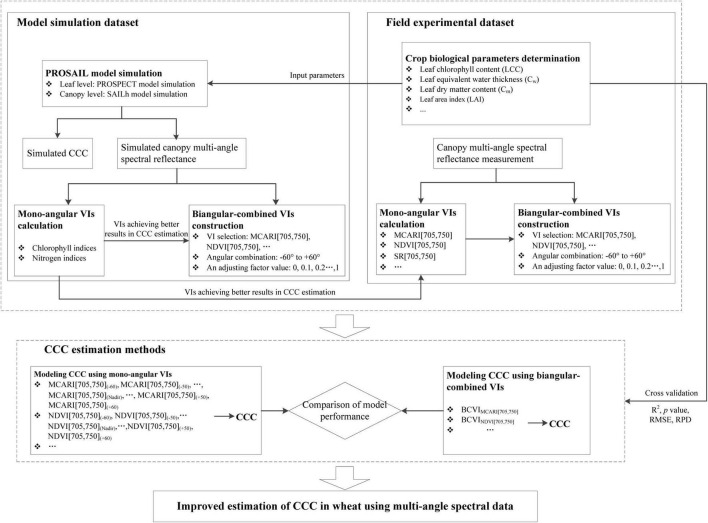
A workflow diagram of canopy chlorophyll content estimation used in this study.

The abilities of mono-angular VIs and BCVIs in assessing CCC were evaluated using two datasets, one was a simulated dataset produced by the PROSAIL model, another was measured from the field campaigns. Linear regression was used to model the relationship between CCC and the two types of indices, while the leave-one-out cross-validation approach was used to validate the models. The coefficient of determination (*R*^2^), *p*-value, and root mean square error (RMSE) were employed as indicators to evaluate the accuracy of estimation models. In addition, the ratio of performance to deviation (RPD), defined as the ratio between the standard deviations of the CCC to predict over RMSE (Richter et al., 2012), was also computed. The prediction ability of the model was interpreted according to the three classes of RPD: RPD > 2 is considered as excellent model performance, 1.4 < RPD < 2 is considered as good model performance, and RPD < 1.4 is considered as unacceptable model performance (Shepherd and Walsh, 2002; Razakamanarivo et al., 2011). The *R*^2^, RMSE, and RPD were calculated as Equations 9–11. For each dataset, VIs that showed the highest *R*^2^ and RPD, and the lowest RMSE with CCC was considered the optimal candidates for predicting CCC. Improvement of CCC estimation was assessed by comparing the estimations of CCC based on mono-angular data and based on multi-angular data. Figure 2 shows the methodology of CCC estimation used in this study.


(9) $${\text{R}}^{2}=\frac{\sum_{i=1}^{n}{\left(\left(y_{mea}^{i}-{\bar{y}}_{mea}\right)\left(y_{est}^{i}-{\bar{y}}_{est}\right)\right)}^{2}}{\sum_{i=1}^{n}{\left(y_{mea}^{i}-{\bar{y}}_{mea}\right)}^{2}\sum_{i=1}^{n}{\left(y_{est}^{i}-{\bar{y}}_{est}\right)}^{2}}$$

(10) $$\text{RMSE}=\sqrt{\sum_{i=1}^{n}{\left(y_{est}^{i}-y_{mea}^{i}\right)}^{2}/n}$$

(11) $$\text{RPD}=\frac{\text{SD}\left(mea\right)}{\text{RMSE}}$$

where *y*_*mea*_ is the measured CCC, $\bar{y}$*_*mea*_* is the average value of measured CCC, ${\bar{y}}_{est}$ is the estimated CCC, ${\bar{y}}_{est}$ is the average value of estimated CCC, *n* is the number of samples, and SD (*mea*) is the standard deviation of measured CCC.

## Results

### Results Based on Model Simulation Data

#### Angular Anisotropy in Spectral Reflectance

**FIGURE 3:**
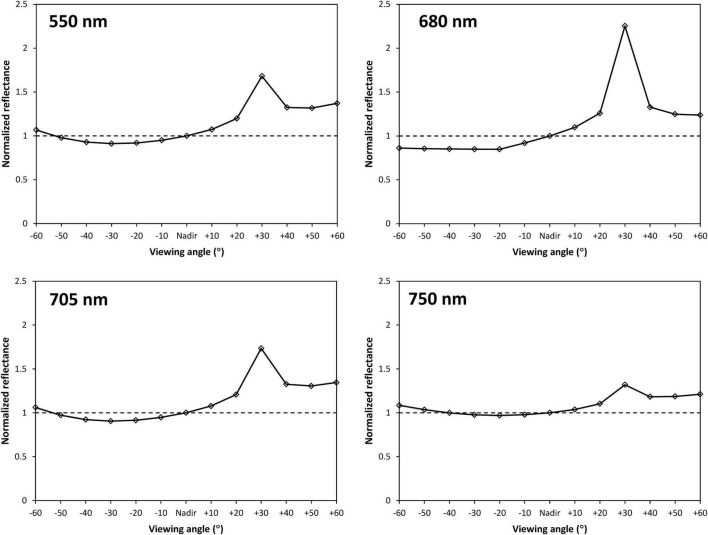
The curves of normalized reflectance at different viewing angles for green (550 nm), red (680 nm), red edge (705 nm), and NIR (750 nm) bands. The viewing angles varied from –60° to +60° with 10° incremental steps, where a positive angle refers to the back-scattering direction, a negative angle refers to the forward-scattering direction. The dash lines indicate normalized reflectance = 1, where the reflectance was measured from the nadir direction.

The curves of normalized reflectance at different viewing angles for green, red, red edge, and NIR bands are shown in Figure 3. It was observed that angular anisotropies in spectral reflectance were pronounced. The reflectance obtained at back-scattering directions was higher than that at the nadir and forward-scattering directions over green to NIR bands, expressing larger normalized reflectance values (R_(θ)_/R_(Nadir)_ > 1). A dominant hot spot with the maximum reflectance appeared at +30° viewing angle at each band, which exactly matched the solar zenith angle in the principal plane. Compared to the back-scattering observations, changes of reflectance obtained from forward-scattering directions tended to be relatively stable. The dark-spot with the minimum reflectance occurred between −20° and −30° viewing angles. Judged by the fluctuations of normalized reflectance values, strong angular anisotropy was observed at chlorophyll absorbance band represented by the 680 nm, whereas weak angular anisotropy appeared in the 550 nm, the 705 nm, and particularly in the 750 nm.

#### Relationship of Mono-Angular Vegetation Indices With Canopy Chlorophyll Content

The linear regression models between the mono-angular VIs and CCC were established, the values of coefficient of determination (*R*^2^) at different viewing observations are shown in Table 4. We found substantial variation in the ability of mono-angular VIs to accurately track the CCC of wheat. In general, the VIs that use bands in red edge and NIR performed better than those with similar formulas, but use bands in red and NIR across all observing angles, such as MCARI[705,750] vs. MCARI, MCARI/OSAVI[705,750] vs. MCARI/OSAVI, TCARI/OSAVI[705,750] vs. TCARI/OSAVI, NDVI[705,750] vs. PSNDa, which further confirmed the promising contribution of red edge bands in improving CCC estimate. Some VIs, however, showed a somewhat weaker relationship with the CCC in particular for the TVI and NRI, with *R*^2^ ranging from 0 to 0.17.

**TABLE 4:** The *R*^2^ values of estimation models between canopy chlorophyll content and VIs at different viewing angles.

Vegetation index	−60	−50	−40	−30	−20	−10	Nadir	+10	+20	+30	+40	+50	+60
PSNDa	0.34**	0.34**	0.35**	0.36**	0.37**	0.37**	0.37**	0.38**	0.38**	0.46**	0.37**	0.35**	0.35**
PSNDb	0.51**	0.49**	0.48**	0.48**	0.48**	0.49**	0.49**	0.50**	0.51**	0.60**	0.52**	0.51**	0.52**
NDVI[705,750]	0.62**	0.65**	0.67**	0.68**	0.69**	0.69**	0.69**	0.70**	0.70**	0.77**	0.67**	0.64**	0.60**
SR[705,750]	0.71**	0.78**	0.82**	0.84**	0.85**	0.86**	0.85**	0.84**	0.83**	0.91**	0.78**	0.74**	0.68**
CI_green_	0.74**	0.80**	0.84**	0.86**	0.88**	0.88**	0.88**	0.87**	0.86**	0.90**	0.81**	0.77**	0.71**
CI_re_	0.75**	0.81**	0.84**	0.86**	0.87**	0.88**	0.87**	0.86**	0.85**	0.90**	0.80**	0.77**	0.72**
MCARI	0.16**	0.15**	0.14**	0.14**	0.14**	0.14**	0.14**	0.14**	0.13**	0.11**	0.15**	0.17**	0.20**
MCARI[705,750]	0.82**	0.87**	0.89**	0.90**	0.91**	0.91**	0.91**	0.91**	0.91**	0.93**	0.88**	0.85**	0.81**
MCARI/OSAVI	0.20**	0.19**	0.19**	0.19**	0.19**	0.19**	0.19**	0.19**	0.18**	0.16**	0.19**	0.21**	0.23**
MCARI/OSAVI[705,750]	0.82**	0.86**	0.88**	0.89**	0.90**	0.90**	0.90**	0.90**	0.89**	0.92**	0.87**	0.85**	0.81**
TCARI	0.24**	0.23**	0.23**	0.23**	0.22**	0.22**	0.21**	0.21**	0.19**	0.16**	0.20**	0.22**	0.24**
TCARI/OSAVI	0.36**	0.38**	0.40**	0.41**	0.42**	0.41**	0.41**	0.39**	0.37**	0.37**	0.35**	0.35**	0.34**
TCARI/OSAVI[705,750]	0.74**	0.81**	0.85**	0.87**	0.88**	0.88**	0.88**	0.87**	0.85**	0.90**	0.80**	0.76**	0.70**
TVI	0.07**	0.10**	0.12**	0.13**	0.14**	0.14**	0.15**	0.15**	0.16**	0.17**	0.14**	0.11**	0.08**
MTVI1	0.32**	0.35**	0.38**	0.39**	0.39**	0.40**	0.39**	0.39**	0.38**	0.36**	0.36**	0.34**	0.30**
REP	0.77**	0.80**	0.83**	0.84**	0.85**	0.85**	0.84**	0.83**	0.81**	0.76**	0.77**	0.76**	0.73**
NDVI_g–b_	0.36**	0.38**	0.41**	0.43**	0.44**	0.45**	0.45**	0.44**	0.44**	0.47**	0.42**	0.40**	0.38**
NRI	NS	NS	NS	NS	NS	NS	NS	NS	NS	NS	NS	0.02*	0.06**
NDDA	0.49**	0.52**	0.53**	0.54**	0.54**	0.54**	0.54**	0.53**	0.51**	0.49**	0.49**	0.48**	0.47**
RVI	0.74**	0.80**	0.84**	0.87**	0.88**	0.88**	0.88**	0.87**	0.86**	0.91**	0.81**	0.77**	0.71**

*Colors correspond to the level of performance, the dark green for large R^2^ and the light green for small R^2^.*

The symbols “**” and “*” indicate canopy chlorophyll content (CCC), and mono-angular VI were significantly correlated with p < 0.01 and p < 0.05, respectively. The NS indicates no significant correlation was found.

**FIGURE 4:**
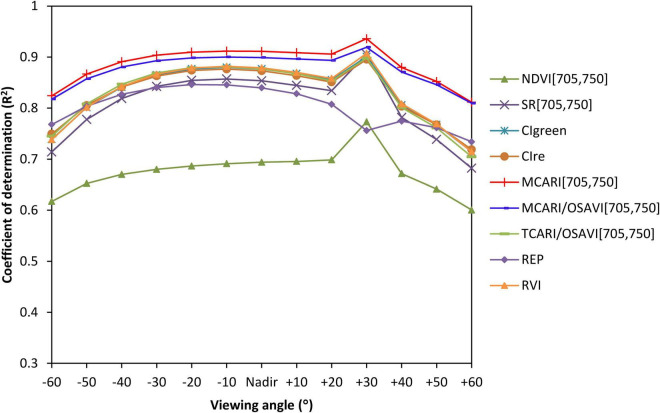
The changing curves of *R*^2^ values for VIs that performed well in canopy chlorophyll content (CCC) estimation at different viewing angles using model simulated data.

Figure 4 shows the performances of VIs that could explain more than 50% variations in CCC (*p* < 0.01) at different viewing angles, i.e., NDVI[705,750], SR[705,750], CI_green_, CI_re_, MCARI[705,750], MCARI/OSAVI[705,750], TCARI/OSAVI[705,750], and REP and RVI, which enabled efficient extraction of the most sensitive mono-angular VIs for CCC determination. We can observe that the coefficient of determination of all VIs exhibited similar trends along with the variety of viewing angles, except for the REP. The higher correlation between the mono-angular VIs and CCC occurred at −30° to +30° observations with the nadir direction included (*R*^2^ ranged from 0.60 to 0.94), while the maximum *R*^2^ appeared at the measurement closest to the hot-spot, which is 30° back-scattering angle in our study. Among all VIs tested, the MCARI[705,750] showed the greatest potential for CCC modeling with *R*^2^ higher than 0.82 for all viewing angles. It gave rise to the most significant correlations with CCC at +30° angle with an *R*^2^ of 0.93 (*p* < 0.01).

#### The Potential of Biangular-Combined Vegetation Indices for Canopy Chlorophyll Content Estimation

**FIGURE 5:**
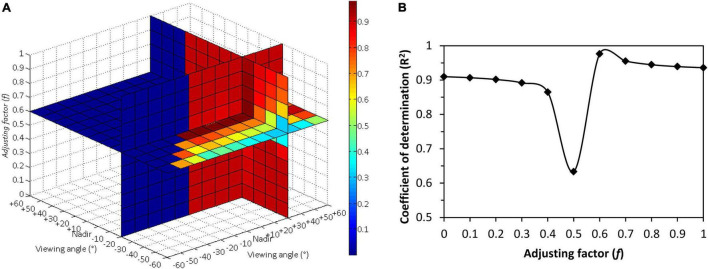
**(A)** The optimum three-dimensional slice map of the coefficients of determination (*R*^2^) for relationship between the canopy chlorophyll content and biangular-combined vegetation indices (BCVI)_MCARI[705,750]_ calculated by the modified chlorophyll absorption ratio index (MCARI)[705,750] at all the possible two-angle observations selected from 13 viewing angles between –60° and +60°, in which an adjusting factor *f* varied from 0 to 1 at a step of 0.1. **(B)** Changing curve of *R*^2^ for relationship between canopy chlorophyll content and BCVI_MCARI[705,750]_ at +30° and –20° angle combination along with variety of *f* values.

The *R*^2^ of the linear estimation model based on the newly developed BCVIs was calculated with respect to the CCC. Table 5 summarized the optimal two-angle combination (i.e., θ1 and θ2), the adjusting factor “*f*” constructed in each best performing BCVI and corresponding maximum *R*^2^ values of CCC modeling. Results indicated that for almost all BCVIs, *R*^2^ reached the peak when indices calculated from reflectance obtained from +30° and −20° or +30° and −30° angle combinations, and at the same time, *f* ranged from 0.6 to 0.7. As expected, the BCVI_MCARI[705,750]_ was found to be advantageous over all the other BCVIs in CCC determination. The combination of θ1 = +30°, θ2 = −20°, *f* = 0.6 was selected from hundreds of angles and adjusting factor combinations, due to its outstanding performance in capturing variations in CCC with *R*^2^ up to 0.98 (Figure 5). Furthermore, it should be noteworthy that the value of *f* appeared to be very significant in affecting the accuracy of CCC modeling at a given most sensitive two-angle combination. As shown in Figure 5B, *R*^2^ of models derived from the BCVI_MCARI[705,750]_ tended to be a bell-shape with increasing *f* values, with minimal *R*^2^ appearing around *f* = 0.5. However, they achieved higher *R*^2^ when *f* varied from 0.6 to 1 compared to *f* changed from 0 to 0.4, implying that the spectral reflectance obtained from back-scattering directions may contribute more than that collected from forward-scattering directions for enhancing CCC estimation in wheat.

**TABLE 5:** The optimal two-angle combination (θ1 and θ2), the adjusting factor *f* constructed in each best performing BCVI, and the corresponding maximum *R*^2^ for canopy chlorophyll content estimation using model-simulated data.

Biangular-combined vegetation index	θ 1	θ 2	*f*	*R* ^2^
BCVI_NDVI[705,750]_	+30	−20	0.6	0.9
BCVI_SR[705,750]_	+30	−20	0.7	0.97
BCVI_CIgreen_	+30	−20	0.7	0.95
BCVI_CIre_	+30	−30	0.7	0.95
BCVI_MCARI[705,750]_	+30	−20	0.6	0.98
BCVI_MCARI/OSAVI[705,750]_	+30	−20	0.7	0.93
BCVI_TCARI/OSAVI[705,750]_	+40	−20	0.6	0.91
BCVI_REP_	+30	−20	0.6	0.93
BCVI_RVI_	+30	−30	0.7	0.96

**FIGURE 6:**
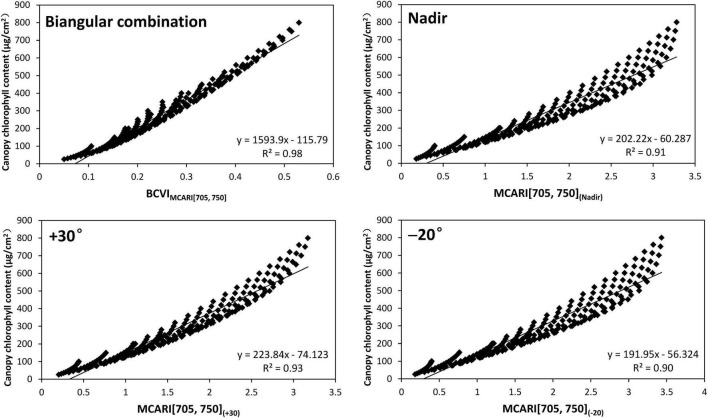
Scatterplots of relationships between canopy chlorophyll content and the MCARI[705,750]_(Nadir)_, MCARI[705,750]_(+30)_, MCARI[705,750]_(–20)_, and BCVI_MCARI[705,750]_ for model simulated dataset.

An important piece of information revealed in Table 5 was that all BCVIs showed better correlations with CCC than the corresponding VIs at any mono-angular observation, even including the most sensitive 30° back-scattering angle, as well as the nadir direction (Table 4). For instance, the BCVI_NDVI[705,750]_ generated the biggest increase in *R*^2^ by 16.9% in comparison of the mono-angular NDVI[705,750]_(+30)_. To further explore how the biangular-combined and mono-angular VIs worked in CCC estimation, the scatterplots of relationships between CCC and the best performing BCVI_MCARI[705,705]_ and mono-angular MCARI[705,750] at the nadir, +30°, −20° viewing angles were taken as an example, as shown in Figure 6. Results demonstrated that on the one hand, the mono-angular MCARI[705,750] at the three different viewing angles all behaved rather more widely scattering against CCC compared to the BCVI_MCARI[705,750]_. On the other hand, the mono-angular MCARI[705,750] reached a saturation level asymptotically when CCC at high values, whereas the BCVI_MCARI[705,750]_ constructed based on +30° and −20° angular combination showed a better trend without a clear saturation; it was strongly and linearly related to the CCC.

### Results Based on Field Experimental Data

#### Model Canopy Chlorophyll Content Using Mono-Angular Vegetation Indices

**FIGURE 7:**
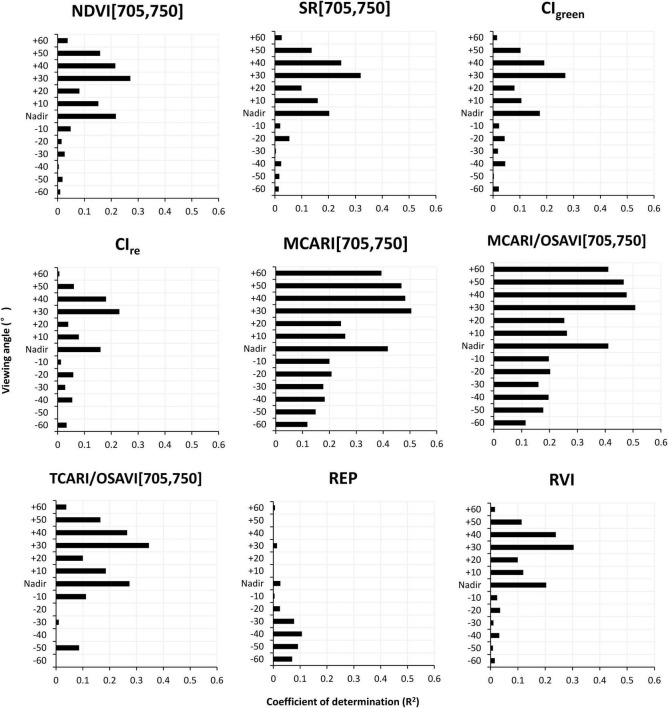
The *R*^2^ of relationship between better performing VIs (shown in Figure 4) and field measured canopy chlorophyll content at different viewing angles.

Nine VI showed in Figure 4 that described the CCC better were tested with the ground truth measurements. Figure 7 shows the results of the relationship between VIs derived from mono-angular spectral reflectance and ground measured CCC. Similar to the results of simulated data, in CCC determination, the +30° angle yielded greater significance than the other angles for all mono-angular VIs, except the REP which only had little sensitivity to the variations in CCC (*R*^2^ ≤ 0.1), making it barely suitable for CCC estimation. The model performances based on the mono-angular MCARI[705,750] and the mono-angular MCARI/OSAVI[705,750] were superior to the others, with comparative and highest *R*^2^ of 0.51 and 0.50, respectively at +30° viewing angle. Apart from the REP, the analogous pattern of *R*^2^ changes for all VIs at different viewing angles was observed: besides the +30° angle, the CCC also showed a better relationship with VIs at both the nadir and +40° directions; interestingly, for the forward-scattering observations, there were two weak peaks with relative high *R*^2^, at −20° and −40° angles, predominating in CCC estimation for most of VIs (SR[705,750], CI_green_, CI_re_, MCARI[705,750], MCARI/OSAVI[705,750], and RVI).

#### Model Canopy Chlorophyll Content Using Biangular-Combined Vegetation Indices

**FIGURE 8:**
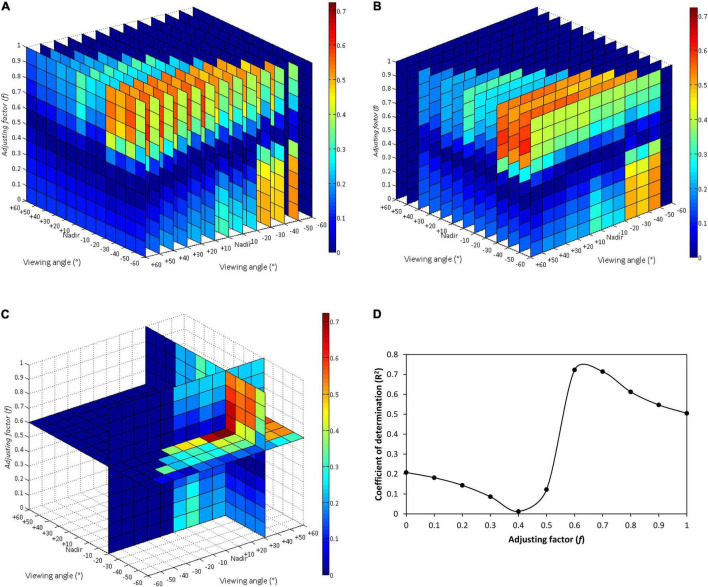
The three-dimensional slice maps of the *R*^2^ for relationship between field measured canopy chlorophyll content and BCVI_MCARI[705,750]_ calculated by the subtraction of MCARI[705,750] at all the possible two-angle observations selected from 13 viewing angles between –60° and +60°, in which an adjusting factor *f* varied from 0 to 1 at a step of 0.1: **(A)** slice map for the first viewing angle (θ1) selection, **(B)** slice map for the second viewing angle (θ2) selection, **(C)** slice map for optimum two-angle and *f* value combination; **(D)** changing curve of *R*^2^ for relationship between field measured canopy chlorophyll content and BCVI_MCARI[705,750]_ at +30° and –20° angle combination along with variety of *f* values.

A series of BCVIs was established with field measured datasets using the same method used in the section “The Potential of Biangular-Combined Vegetation Indices for Canopy Chlorophyll Content Estimation,” and were examined the linearity to CCC. The BCVI_MCARI[705,750]_ was chosen as an example to illustrate the process of the selection of three parameters (θ1, θ2, and *f*) composing in the BCVI. From the slice maps shown in Figure 8, *R*^2^ varied intensely with changing of different combinations of MCARI[705,750] values at two viewing angles. The BCVI_MCARI[705,750]_ that was calculated by the subtraction of MCARI[705,750] at +30° and −20° angular combination with *f* = 0.6 as an adjusting factor stood out among all combinations, with the soundest *R*^2^ for CCC estimation (*R*^2^ = 0.72), which was consistent with the previous results of dataset simulated by PROSAIL model. Meanwhile, the changing curve of *R*^2^ values exhibited a slightly different shape but a similar trend, with the simulated BCVI_MCARI[705,750]_ (Figure 5B), expressing a more striking contrast between *f* of 0 to 0.3 and *f* of 0.6 to 1 (Figure 8D). This result put emphasis on the greater role of spectral information extracted at back-scattering directions than that at forward-scattering directions, in CCC determination when using the field measured data, in comparison to the simulated data. As for the other seven BCVIs, similar patterns of slice maps and *R*^2^ changing curves occurred (not shown for brevity). We found that the best BCVIs for investigating changes in CCC was also generated at +30° and −20° or +30° and −30° angle combination, in which *f* was around 0.6 to 0.7, with *R*^2^ of 0.34 to 0.71 (Table 6).

**TABLE 6:** The optimal two-angle combination (θ1 and θ2), the adjusting factor *f* constructed in each best performing biangular-combined vegetation indices (BCVI), and the corresponding maximum *R*^2^ for CCC estimation using field measured data.

Biangular-combined vegetation index	θ 1	θ 2	*f*	*R* ^2^
BCVI_NDVI[705,750]_	+30	−30	0.6	0.41
BCVI_SR[705,750]_	+30	−30	0.7	0.42
BCVI_CIgreen_	+30	−20	0.7	0.38
BCVI_CIre_	+30	−30	0.7	0.34
BCVI_MCARI[705,750]_	+30	−20	0.6	0.72
BCVI_MCARI/OSAVI[705,750]_	+30	−20	0.6	0.71
BCVI_TCARI/OSAVI[705,750]_	+30	−20	0.6	0.45
BCVI_RVI_	+30	−20	0.7	0.38

#### Comparison of Performances of Biangular-Combined Vegetation Indices and Mono-Angular Vegetation Indices

**FIGURE 9:**
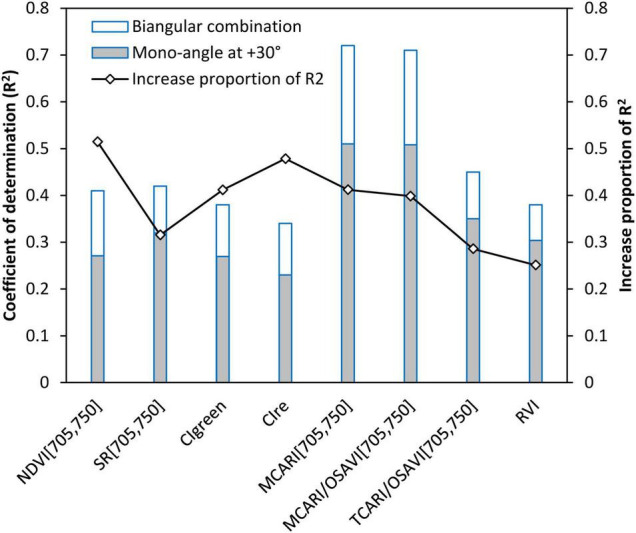
Comparisons of *R*^2^ values of relationships between biangular-combined vegetation indices (VIs) vs. canopy chlorophyll content and mono-angular VIs at +30° viewing angle vs. canopy chlorophyll content.

To explore what degree the multi-angular viewing capability of spectra can contribute to the improved CCC assessment of wheat compared to the mono-angular observations, we analyzed the performances of the newly developed BCVIs and the corresponding mono-angular VIs at the most sensitive +30° angle (Figure 9). The result revealed that the BCVIs showed a clear increase in *R*^2^ by 25.1–51.4%, as compared to the mono-angular VIs, and were proven to be more effective and suitable in modeling CCC. The most significant improvement was observed in the comparison between indices of BCVI_NDVI[705,750]_ and NDVI[705,750]_(+30)_. As previously explained, the BCVI_MCARI[705,750]_ and MCARI[705,750]_(+30)_ showed the strongest correlation with CCC among all BCVIs and mono-angular VIs, respectively, but the BCVI_MCARI[705,750]_ further improved the CCC estimation accuracy by 41.2%.

**FIGURE 10:**
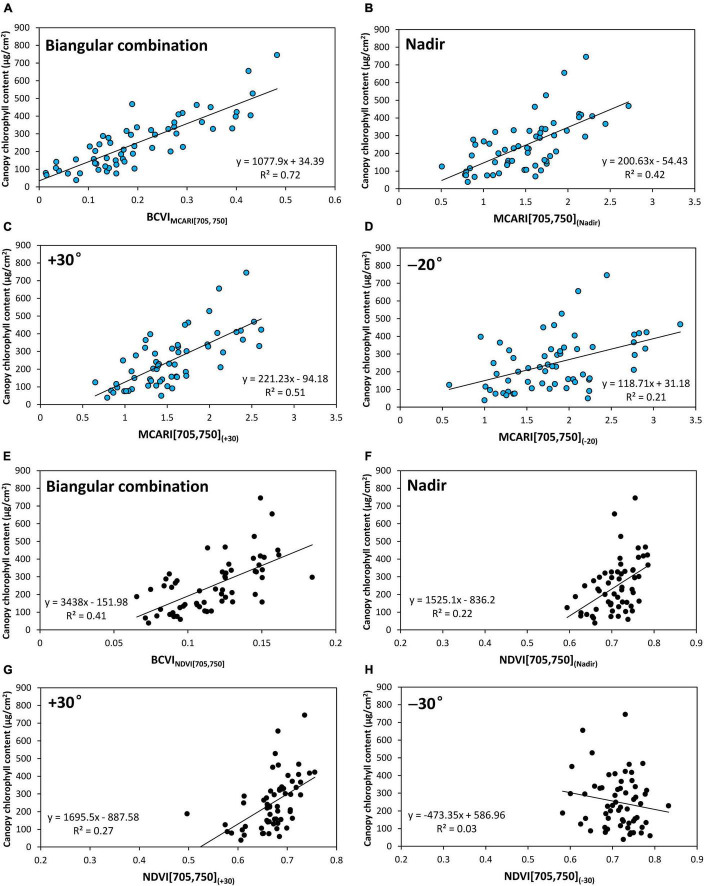
Scatterplots of relationships between canopy chlorophyll content and BCVIs and the corresponding mono-angular VIs based on MCARI[705,750] and normalized difference vegetation index (NDVI)[705,750] for filed experimental datasets. The blue points **(A–D)** represent the BCVI_NDVI[705,750]_, MCARI[705,750]_(Nadir)_, MCARI[705,750]_(+30)_, and MCARI[705,750]_(–20)_, respectively; the black points **(E–H)** represent the BCVI_NDVI[705,750]_, NDVI[705,750]_(Nadir)_, NDVI[705,750]_(+30)_, and NDVI[705,750]_(–30)_, respectively.

We further plotted the scatterplots of BCVIs and mono-angular VIs at the nadir, +30° and −20° or −30° viewing angles based on the MCARI[705,750] and NDVI[705,750] against CCC (Figure 10). For the two VIs, the BCVIs were characterized by less scattered relationships with CCC compared to their mono-angular counterparts derived from the nadir, +30° and −20°/−30° directions, in particular, for the BCVI_MCARI[705,750]_. In consistent with the simulated result, as illustrated in Figure 6, BCVI_MCARI[705,750]_ behaved linearly with CCC, with the scatter points evenly distributed around the fitting line, clearly depicting the dynamic changes of CCC (Figures 10A–D). As can be seen in Figures 10E–H, the sensitivities of NDVI[705,750]_(Nadir)_ and NDVI[705,750]_(+30)_ were most affected by high values of CCC, showing a saturation effect when CCC exceeded 400 μg/cm^2^. However, the BCVI_NDVI[705,750]_ improved the linearity and reduced the saturation limit of mono-angular NDVI[705,750] at the three viewing angles to a great extent.

#### Testing Canopy Chlorophyll Content Estimation Models

**FIGURE 11:**
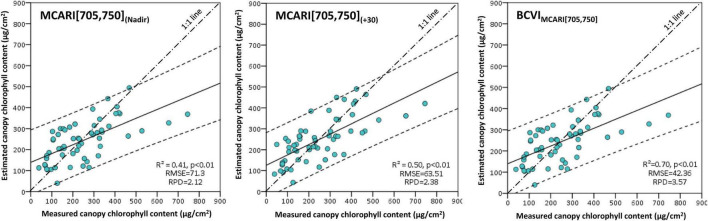
Scatterplots between measured canopy chlorophyll content and estimated canopy chlorophyll content based on the MCARI[705,750]_(Nadir)_, MCARI[705,750]_(+30)_, and BCVI_MCARI[705,750]_. The solid lines indicate the regression fitting lines, the dash lines indicate the 95% confidence intervals of prediction, the long and short dash lines indicate 1:1 lines.

The mono-angular MCARI[705,750] at the nadir and +30° viewing angles, as well as the BCVI_MCARI[705,750]_ were chosen to test the potential of these VIs in predicting the CCC by the means of cross-validation since they were proven to be reliable in CCC estimation both for simulated data and experimental data. The predictions of the three indices against the ground measured CCC were plotted in Figure 11. The CCC was well-predicted by MCARI[705,750]_(+30)_ and MCARI[705,750]_(Nadir)_, with RPDs larger than 2.12 and scattered in both plots fell into the 95% confidence intervals. The MCARI[705,750]_(+30)_ generated relative higher accuracy than the MCARI[705,750]_(Nadir)_ and *R*^2^ of 0.50 (*p* < 0.01) were observed for the measured dataset with RMSE of 63.51. In comparison with the two mono-angular MCARI[705,750], we found a more consistent agreement between CCC values measured in the field and those estimated by the new derived BCVI_MCARI[705,750]_, with coefficient of determination of 0.70 (*p* < 0.01), RMSE of 42.36, and RPD of 3.57. The results suggested that the BCVI_MCARI[705,750]_ at +30° and −20° angle combination performed better and could be more preferable than the conventional nadir direction approach to remote sensing CCC in wheat.

## Discussion

In this study, we estimated crop CCC using simulated multi-angle remote-sensing data produced with the PROSAIL model and canopy multi-angle hyperspectral reflectance measured from the field of winter wheat. We developed the BCVIs by coupling, not only spectral but also angular information, and compared the performances of these BCVIs with the corresponding mono-angular VIs to evaluate whether the CCC estimate could be improved from multi-angle observations. From the characteristics of multi-angle spectral reflectance, angular anisotropy was greatly different at the chlorophyll absorbance and canopy reflective bands. This was mainly due to the discrepancy of the contrast between shadowed and illuminated canopy components at both two types of bands, which resulted from viewing and illumination geometry and the sensor’s field of view (Kollenkark et al., 1982; Galvao et al., 2009). In the case of crop canopy, the reflectance at the green, red edge, and particular NIR bands had low absorbance but highly reflective values. The contrast at these bands was effectively reduced because of multiple scattering processes (Sandmeier et al., 1998), compared to the red absorbance band, weakening the expression of angular anisotropy as demonstrated in Figure 3. However, for all spectral bands tested, reflectance exhibited higher values at back-scattering directions than forward-scattering directions in the solar principal plane, primarily because more and more fractions of illuminated leaf surfaces were viewed by the sensor, along with its rotation from the side facing away from the sun to the side facing into the sun. As confirmed by the study of Sandmeier et al. (1998), the well-illuminated vegetation canopy would be less vulnerable to the shadow effect, which led to more signals from leaves that can be detected by the spectral reflectance measured from the back-scattering directions. Indeed, our results also indicated that almost all mono-angular VIs were more closely related to the CCC at the back-scattering directions than the forward-scattering directions for datasets of both model simulation and ground measurements (Table 4 and Figure 7).

Angular effect presented in VIs can either be regarded as a superfluous uncertainty for vegetation parameters estimation or as a source of additional information that enhances the accuracy of the parameter assessment at canopy scale (Verrelst et al., 2008). In this study, the CCC estimation based on 30° back-scattering spectral data led to an improvement, as compared to the conventional nadir data. This can be attributed to the reduction of soil background impact which is mostly contained at the nadir observation. A similar result was also observed in the studies of He et al. (2016), Kong et al. (2017), and Inoue et al. (2008), who studied the improvements of physiological parameters estimation for crops (e.g., canopy nitrogen content, canopy carotenoid content, and photosynthetic efficiency) based on multi-angle hyperspectral remote-sensing. However, it is important to notice that the estimation of CCC improved even further than mono-angular VIs at any viewing angle when the multi-angular information was added, especially in the case of the ground truth measurement. There are several explanations for having excellent behavior. In this study, we developed the BCVIs based on existing VIs proposed for vegetation chlorophyll and nitrogen estimates, by applying an iterative optimization approach, since it can search for the optimal biangular combination from all the available viewing angles and the most suitable adjusting factor value used. The result showed that the angular combination composing of the best BCVI came close to the positions of hot-spot and dark-spot in the solar principal plane. For one thing, vegetation canopy is a non-Lambert in nature, multi-angle spectral reflectance can capture the uneven scattering of sunlight by vegetation, reaching the maximum at the hot-spot direction, and the lower value at the dark-spot position as suffering from the shadow effect. Because the composition of shadowed and illuminated canopy components is highly dependent on LAI, leaf orientation distributions, and other structural properties (Stagakis et al., 2010), the striking difference of VI values between hot spot and dark spot, that adjusted by the value of “*f*,” not only facilitated to provide more information on chlorophyll than the VI at the solely mono-angle, but more importantly, made the information on three-dimensional vegetation structures prominent when the BCVI was used for tracking the changes of chlorophyll content at canopy scale. Additionally, our work also put heavy emphasis on the importance of the value of the adjusting factor “*f*” in the formula of BCVI for promoting the CCC estimation, but it has not yet received widespread attention from researchers working on quantifying CCC using multi-angle spectral data. We selected the value of “*f*” systematically, from 0 to 1 with a step of 0.1, and concluded that the BCVIs performed best when *f* was 0.6 or 0.7, which implied that the contribution of VIs around the hot-spot angle was greater than that of VIs around the dark-spot angle. This allowed more signals from sunlit leaves to be included, consequently, increasing the quality of vegetation biochemical parameters (e.g., chlorophyll) reflectance contained. For another, compared the reflectance spectra measured at a single viewing angle, the BCVIs derived from multi-angle spectral data may improve the CCC inversion by including additional information on leaf chlorophyll at different vertical layers within canopies (Huang et al., 2011).

A high amount of studies have demonstrated that many VIs are prone to saturation with increasing vegetation biological variables (Broge and Leblanc, 2001; Haboudane et al., 2004; Wu et al., 2008). This limitation of saturation was also found in the analysis of the relationship between mono-angular VIs and CCC for overall datasets in our study (Figures 6, 10), including the best performing MCARI[705,750], which may restrict the reliability of their use in monitoring dense canopies with higher CCC. Nevertheless, the saturation phenomenon was overcame in large part by introducing the corresponding VI obtained from another viewing angle to construct the BCVI; results of the newly developed BCVI showed better linearity and higher accuracy (e.g., *R*^2^ = 0.98 and 0.72 for BCVI_MCARI[705,750]_ for PROSAIL-simulated and filed experimental datasets, respectively), and features the scattered display more intensively, along with the changing of CCC than those for any mono-angular VI. This is another reason why the BCVIs held more promising potential in CCC assessment.

For the simulated and experimental datasets, the accuracy of CCC estimation was all improved when using the BCVIs we proposed. However, there were evident discrepancies in the magnitude of improvement observed between both datasets. The main possibility was that the PROSAIL model was not capable of adequately reproducing the real physiological and morphological conditions of the wheat canopy that we used in this study. For instance, more and more studies have demonstrated that the vertical distribution of leaf biochemical variables (e.g., chlorophyll and nitrogen) was non-uniform within plant canopy (Ciganda et al., 2012; He et al., 2020), leading to different contributions of the vertical layers to canopy spectra (Li et al., 2015). Moreover, apart from leaves, the presence of other canopy components, such as wheat spikes and plant stems, would also have an impact on canopy reflectance (Haboudane et al., 2004; He et al., 2019) and as a result the quality of extracted crop CCC. But these factors are not taken into account in the PROSAIL model. In addition, to focus on exploring the sensitivities of different mono-angular VIs and the developed BCVIs to CCC variation, LCC and LAI were set as free variables, while the other vegetation parameters were set to fixed constants at their respective mean values when conducting the canopy multi-angled spectral reflectance simulation. Nevertheless, this vegetation parameter setting may become a primary source causing the differences with results of field measurements, since many parameters (e.g., leaf carotenoid, water, and fresh and dry matter), besides LCC and LAI, were continually changing with the growth of crops and the implementation of different agricultural managements in reality. Due to large improvements for field measurement dataset were obtained, our results establish the confidence in the use of the BCVIs we developed for crop CCC modeling in the future practical application. Nowadays, some space-borne sensors have been specifically designed to collect data from multi-angle observations (Barnsley et al., 2004; Roosjen et al., 2018), solid coupling of radiative transfer model, and multi-angle spectral information will be key to the successful assessment of chlorophyll content at canopy scale. Because the hot-spot reflectance is difficult to be adequately acquired and, thus, they might be interpolated from the adjacent measurements, future studies will be needed to further validate whether the BCVIs derived from satellite data could provide more accurate crop CCC estimation.

## Conclusion

The CCC is a measure of photosynthetic potential at the canopy level and was retrieved as the product of leaf chlorophyll and LAI. Unlike previous analyses that mainly focused on establishing VI from mono-angular remote sensing data, we proposed a new method of developing the BCVIs for high-throughput estimation of crop CCC using canopy multi-angle observations. The BCVI was calculated by the subtraction of chlorophyll-sensitive VI values, computing from reflectance measured around hot-spot and dark-spot positions, with 0.6 or 0.7 as an adjusting factor. This algorithm involved both leaf chlorophyll and canopy structural information making the BCVIs derived from multi-angle spectral reflectance more effective when compared to solely using corresponding mono-angular VIs at arbitrary viewing angle for assessing CCC across PROSAIL model-simulated dataset and field experimental dataset. The MCARI[705,750] was proven to be the best mono-angular VI among previously published VIs tested, +30° back-scattering angle produced better performing VI than the nadir direction. However, as they were equally subject to the saturation limit with increasing of CCC based on the results, we developed the BCVI_MCARI[705,50]_ at +30° and −20° angle combination, formulated as 0.6×*MCARI*[705,750]_(+ 30)_-0.4×*MCARI*[705,750]_(−20)_ where +30° and −20° angles were the closest measurements to the hot-spot and dark-spot positions in this study. We found that the BCVI_MCARI[705,750]_ was not only resistant to the saturation effect, but also exhibited the highest sensitivity to CCC variation over all datasets. Our results demonstrate that the BCVI that taking spectral and angular information into account could substantially improve the estimation of crop CCC, and consequently offer more accurate information to understand crop’s phenotypic trait across growth stages and their response to environmental changes in agro-ecosystem.

## Data Availability Statement

The raw data supporting the conclusions of this article will be made available by the authors, without undue reservation.

## Author Contributions

WK processed and analyzed the simulated and experimental datasets, and wrote sections of the manuscript. WH, CL, and LT guided the data analysis and provided suggestions for the study. LM, JG, and XZ designed the experiment and were involved in the ground data collection. RC provided the suggestions, and revised and edited the manuscript. All authors contributed to manuscript revision, read, and approved the submitted version.

## Conflict of Interest

The authors declare that the research was conducted in the absence of any commercial or financial relationships that could be construed as a potential conflict of interest.

## Publisher’s Note

All claims expressed in this article are solely those of the authors and do not necessarily represent those of their affiliated organizations, or those of the publisher, the editors and the reviewers. Any product that may be evaluated in this article, or claim that may be made by its manufacturer, is not guaranteed or endorsed by the publisher.
